# Impact of Depression on Irritable Bowel Syndrome (IBS)-Associated Gastrointestinal Symptoms: A Cross-Sectional Study in Makkah City, Saudi Arabia

**DOI:** 10.7759/cureus.61118

**Published:** 2024-05-26

**Authors:** Nasser Al Shanbari, Salah M Bakry, Muath Alzahrani, Muhanna M Almatrafi, Abdullah S Alshanbari, Azzam M Bin Laswad, Faeqah Alharbi, Hazem Alarrafi, Abdulrahman Alnabati, Ayman Alsaedi, Mokhtar Shatla

**Affiliations:** 1 Department of Medicine and Surgery, College of Medicine, Umm Al-Qura University, Makkah, SAU; 2 Department of Community Medicine, College of Medicine, Umm Al-Qura University, Makkah, SAU

**Keywords:** school students, schoolteachers, prevalence, irritable bowel syndrome, depression

## Abstract

Background

Irritable bowel syndrome (IBS) is a functional gastrointestinal chronic disorder associated with symptoms such as abdominal pain, diarrhea, and constipation. One of the factors that could affect the pathogenesis of IBS is depression, a common psychological disorder that causes social and physical disability and affects productivity. A number of Saudi teachers were found to have depression, which was linked with multiple risk factors including chronic illnesses. However, there is limited data that exhibits the association between IBS and depression, specifically. Therefore, our study aims to determine the impact of depression on IBS-associated gastrointestinal symptoms in Makkah City schools, Saudi Arabia.

Methods

In this cross-sectional study, we used two validated scales and translated them into Arabic and then we distributed them to our targeted population. Our sample size was determined to be 383 but we succeeded in recruiting 477 participants in our study. Data were statistically analyzed using the statistical software Statistical Package for Social Sciences (SPSS), version 23.0 (IBM Corp., Armonk, NY).

Results

Generally, participants who demonstrated mild levels of Patient Health Questionnaire-9 (PHQ-9) depression scale corresponded significantly with minimal/mild and moderate levels of Gastrointestinal Symptom Rating Scale-IBS (GSRS-IBS) scores (n = 85 and 76, respectively; *p* ˂ 0.001), while participants who scored moderately on the PHQ-9 depression scale corresponded significantly with a severe level of GSRS-IBS scores (n = 29; *p* ˂ 0.001).

Conclusion

Our study found a significant association between different levels of depression and IBS among participants with a positive history of IBS. Further studies about the prevalence of IBS, depression, and the nature of their relationship are strongly recommended, in addition to the necessity of a suicide risk assessment for those with severe depression.

## Introduction

Irritable bowel syndrome (IBS) is a functional gastrointestinal chronic disorder that is considered to be the most common disorder seen at gastroenterology clinics [1]. It is associated with abdominal pain, diarrhea, constipation, or a combination of all three. It affects 11% of the global population [2]. IBS has a complex pathophysiology that has numerous explanations for its pathogenesis, such as altered mobility of the bowel, visceral hypersensitivity, an imbalance of neurotransmitters, infection, inflammation, and psychosocial factors [3]. IBS is diagnosed by using the Romell criteria, which are symptom-based criteria [4]. IBS can be managed by dietary modifications and pharmacotherapy [5]. One of the factors that can affect the motor function of the small bowel and colon and play a role in the pathogenesis of IBS is depression [3], a common psychological disorder that leads to social and physical disability and affects productivity. Depression affects 322 million people around the world [6]. Common depression symptoms are more likely to be somatic, such as fatigue, insomnia, headaches, and chronic pain, rather than involving feelings of sadness or depression [7]. Depression is diagnosed according to the *Diagnostic and Statistical Manual of Mental Disorders*, Fifth Edition (DSM-5), and can be managed by psychotherapy and pharmacotherapy [7,8]. A study has revealed that up to 60% of IBS patients have major psychosocial problems [9]. It was found that 30.4% of schoolteachers in Saudi Arabia have moderate to moderately severe depression [10]. Many risk factors have been linked with depression among teachers but there are limited studies that assess the association between IBS and depression specifically [11]; thus, our study aims to determine the impact of depression on IBS-associated symptoms in Makkah City schools, Saudi Arabia, and to find out the association between depression and IBS among schoolteachers and students.

## Materials and methods

Study design and ethical consideration

This cross-sectional study used an electronic survey made on Google Forms. It was conducted in June 2022 after our proposal obtained ethical approval from the biomedical ethics committee at the College of Medicine, Umm Al-Qura University, Makkah, Saudi Arabia, with approval number HAPO-02-K-012-2022-05-1080. The procedure was conducted according to the principles of the Declaration of Helsinki principles.

Study criteria

Male and female schoolteachers and students who were residents of Makkah City were included in this study. We excluded those under 15 years old to minimize the misunderstanding of the survey and filling it incorrectly. In addition, we excluded those who were physically ill and incapable of communicating with field researchers and those who refused to participate in this study.

Sample size calculation

According to the Saudi General Authority for Statistics, the number of our targeted population was more than 100000. We calculated our sample size using Epi Info software (version 2.1) (Centers for Disease Control and Prevention, Atlanta, GA) [12]. The minimum sample size was 383, with a perception of 5% and a confidence interval of 95%. We succeeded in distributing our survey to 480 of the study population. However, only 477 agreed to participate in the survey.

Study tool

The survey was obtained from previously published studies [13,14]. The opportunity to contact us for any problems or questions was mentioned at the beginning of the questionnaire, in addition to the consent that was obtained from all the participants. The study questionnaire was divided into three parts: the first part included all the demographic questions and the prevalence of IBS among our study population; the second part aimed to assess the gastrointestinal symptoms of IBS using the Gastrointestinal Symptom Rating Scale-IBS (GSRS-IBS) [14]; and the third part screened for depression symptoms among the population using Patient Health Questionnaire-9 (PHQ-9) [13].

Statistical analysis

We extracted the data and coded it using Microsoft Office Excel (Microsoft Corporation, Redmond, WA), before transferring it into the statistical software Statistical Package for Social Sciences (SPSS), version 23.0 (IBM Corp., Armonk, NY). The p value for statistical significance would be less than 5%. The factors affecting students and the degree of depression were examined using descriptive statistics that relied on frequency ranges and percentage distributions.

## Results

We electronically interviewed 477 school students and teachers from Makkah City schools. Table 1 provides demographic information about the participants. Most participants were under 18 years of age (n = 229; 48%), followed by those aged between 18 and 30 years (n = 203; 42.6%). There was a near-equal representation of male and female participants, with a slight predominance of females (n = 247; 51.8%). At the same time, single participants were represented far more than married participants (n = 425; 89.1%) because most of the survey participants were students. Most participants were Saudis (n = 441; 92.5%). Moreover, students responded at a higher rate than teachers (n = 425; 89.1%). Most of the participants had a monthly income of over 10000 Saudi Arabian Riyal (SAR), followed by 5000-10000 SAR per month (n = 188; 39.4%) and (n = 163; 34.2%), respectively. Most participants were not smokers (n = 439; 92%), and most had no chronic diseases other than IBS (n = 359; 75.3%). Asthma afflicted 10.3% of participants, while 9.6% had other health conditions.

**Table 1:** Participants' social-demographical profiles SAR: Saudi Arabian Riyal

Category		Number of respondents (%)
Age groups	˂ 18	229 (48.0%)
	18-30	203 (42.6%)
	31-40	16 (3.4%)
	41-50	23 (4.8%)
	51-60	3 (0.6%)
	˃ 60	3 (0.6%)
Gender	Male	230 (48.2%)
	Female	247 (51.8%)
Social status	Single	425 (89.1%)
	Married	52 (10.9%)
Nationality	Saudi	441 (92.5%)
	Non-Saudi	36 (7.5%)
Current occupation	Students	425 (89.1%)
	Teachers	52 (10.9%)
Income (SAR)	˂ 5000	126 (26.4%)
	5000-10000	163 (34.2%)
	˃ 10000	188 (39.4%)
Smoking status	Yes	38 (8.0%)
	No	439 (92.0%)
Chronic diseases among participants	Cardiovascular	4 (0.8%)
	Diabetes	11 (2.3%)
	Hypertension	8 (1.7%)
	Asthma	49 (10.3%)
	Other	46 (9.6%)
	None	359 (75.3%)

**Figure 1:**
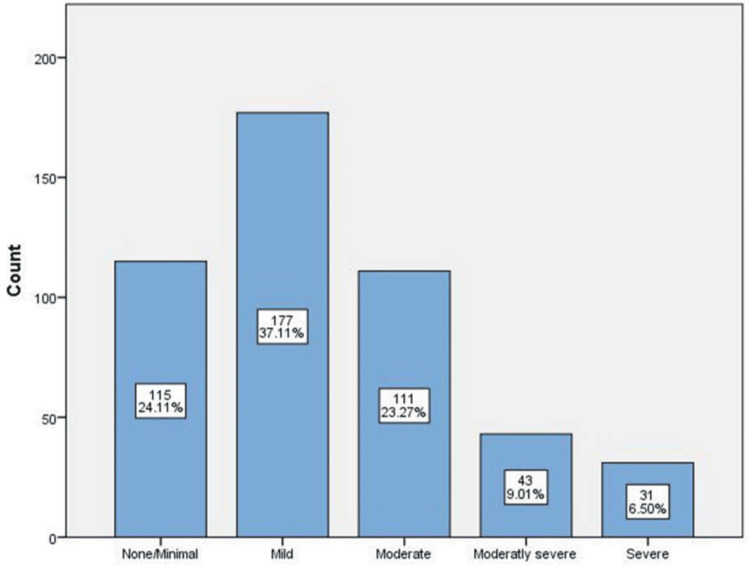
Frequency of depression among participants

Regarding PHQ-9 depression scores, most participants had mild depression scores (n = 177; 37.11%), followed by those with scores of zero or low scores (n = 115; 24.11%) and those with moderate scores (n = 111; 23.27%). However, 9.01% of participants had moderately severe depression scores (n = 43), followed by those with severe depression scores (n = 31; 6.50%) (Figure 1).

**Figure 2:**
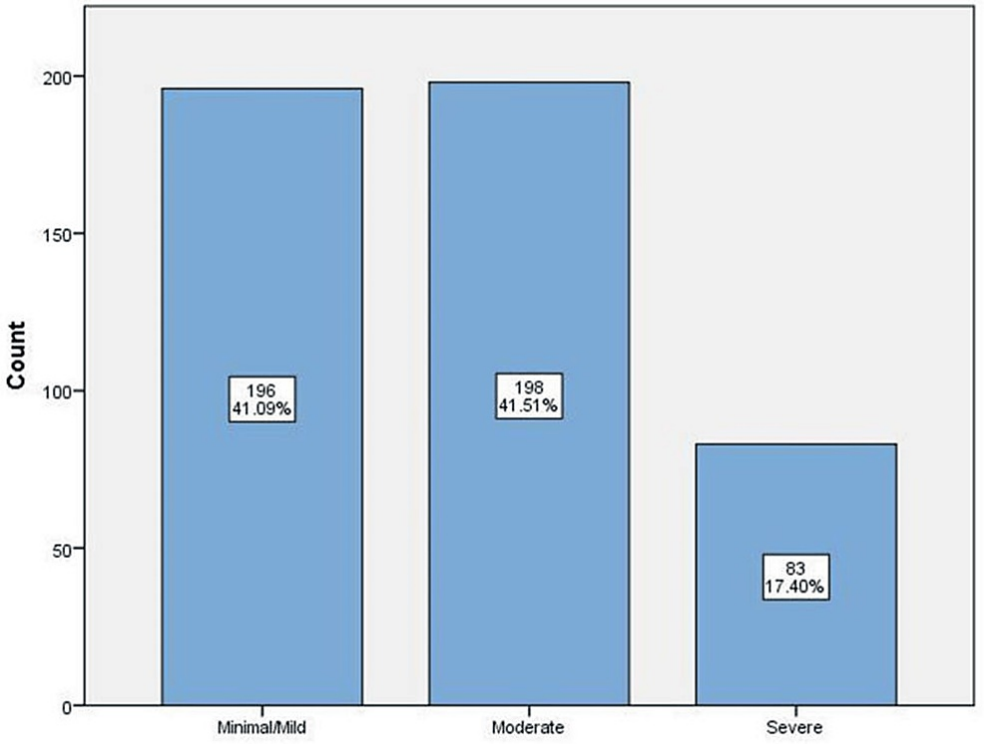
Frequency of IBS symptoms among participants IBS: irritable bowel syndrome

GSRS-IBS scores are shown in Figure 2. There is a similar representation of those with minimal or mild scores and those with moderate scores (n = 196; 41.09% and n = 198; 41.51%, respectively. Participants with severe scores were also represented (n = 83; 17.40%).

The association between participants’ GSRS-IBS scores and PHQ-9 (depression) scores and their demography is shown in Table 2. Participants under 18 corresponded significantly with minimal/mild and moderate score levels of the GSRS-IBS score (n = 108 and 88, respectively; p = 0.060). In contrast, those aged between 18 and 30 showed significant variation, with a severe score level (n = 36; p = 0.060). No significant association was demonstrated between participants’ PHQ-9 depression scores and their age groups. Male participants corresponded significantly with minimal/mild GSRS-IBS score levels (n = 106; p = 0.001), while females corresponded significantly with moderate and severe score levels (n = 112 and 53, respectively; p = 0.001). In contrast, males corresponded significantly with none/minimal and mild scores on the PHQ-9 depression scale (n = 80 and 90, respectively; p ˂ 0.001). Regarding social status, single participants were significantly associated with all GSRS-IBS score levels (p = 0.002). However, no statistically significant association was found between social status and the PHQ-9 depression scale. Surprisingly, compared to teachers, student participants showed a more significant association with all scores on the GSRS-IBS scale (p = 0.001). Additionally, students and teachers showed no significant association with the PHQ-9 depression scale. Furthermore, no significant association was shown to exist between students or teachers and the PHQ-9 depression scale. Participants with no previous chronic diseases showed a significant association with both the GSRS-IBS and PHQ-9 depression scales (p = 0.011 and p ˂ 0.001, respectively).

**Table 2:** Association between GSRS-IBS scores and depression scales in relation to participants’ social-demographical profiles (n = 477) The data in this table are explained in frequencies (N) *P > 0.05 (significant) GSRS-IBS: Gastrointestinal Symptom Rating Scale-Irritable Bowel Syndrome, SAR: Saudi Arabian Riyal

Category		GSRS-IBS			P-value	Depression					P-value
		Minimal/Mild	Moderate	Severe		None-minimal	Mild	Moderate	Moderately severe	Severe	
Age groups	˂ 18	106	88	35	0.060*	64	79	50	22	14	0.538
	18-30	82	85	36		38	79	52	20	14	
	31-40	3	9	4		2	8	5	0	1	
	41-50	3	14	6		7	10	3	1	2	
	51-60	0	2	1		2	1	0	0	0	
	˃ 60	2	0	1		2	0	1	0	0	
Gender	Male	114	86	30	0.001*	80	90	42	13	5	˂0.001*
	Female	82	112	53		35	87	69	30	26	
Social status	Single	186	166	73	0.002*	103	153	98	42	29	0.260
	Married	10	32	10		12	24	13	1	2	
Nationality	Saudi	180	183	78	0.826	109	163	105	37	27	0.243
	Non-Saudi	16	15	5		6	14	6	6	4	
Current occupation	Students	187	168	70	0.001*	101	158	97	42	27	0.418
	Teachers	9	30	13		14	19	14	1	4	
Income (SAR)	˂ 5000	49	48	29	0.251	35	37	28	13	13	0.176
	5000-10000	74	65	24		36	65	34	17	11	
	˃ 10000	73	85	30		44	75	49	13	7	
Smoking status	Yes	16	14	8	0.762	13	10	10	1	4	0.191
	No	180	184	75		102	167	101	42	27	
Chronic diseases among participants	Cardiovascular	0	1	3	0.011*	0	2	0	0	2	˂0.001*
	Diabetes	2	5	4		5	2	0	1	3	
	Hypertension	4	3	1		2	1	1	2	2	
	Asthma	15	27	7		9	17	10	8	5	
	Other	17	16	13		10	12	14	4	6	
	None	158	146	55		89	143	86	28	13	

**Figure 3:**
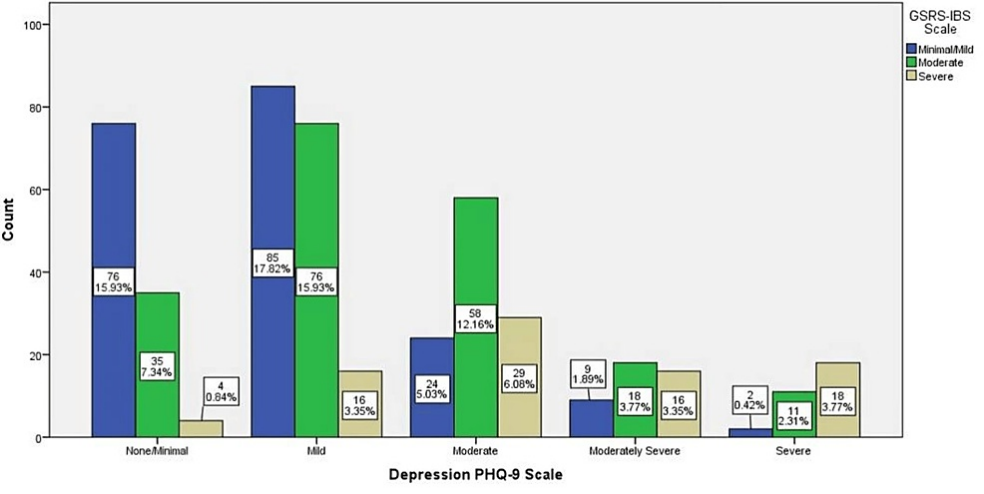
The association between participants' GSRS-IBS scores and PHQ-9 depression scale GSRS-IBS: Gastrointestinal Symptom Rating Scale-Irritable Bowel Syndrome, PHQ-9: Patient Health Questionnaire-9 (PHQ-9)

The association between the GSRS-IBS score level and the PHQ-9 depression scale is shown in Figure 3. Participants with low scores on the PHQ-9 depression scale corresponded significantly with minimal/mild and moderate levels of GSRS-IBS (n = 85 and 76, respectively; p ˂ 0.001), while participants with a moderate score on the PHQ-9 depression scale corresponded significantly with a severe level of GSRS-IBS (n = 29; p ˂ 0.001).

## Discussion

This study, conducted on schoolteachers and students in Makkah City, aimed to determine the impact of depression on individuals with a previous history of IBS. It found that 41.51% of participants had a history of moderate IBS symptoms, with female participants having more symptoms than male participants. Regarding age groups, most of the respondents with moderate IBS history were between 18 and 30 years old, and mostly single, with the majority of them being students, which is due to the fact that most of the survey respondents were students.

The prevalence of IBS was reported at Jouf University as 29.3% among medical and non-medical students and only 16.4% among non-medical students. Another dissimilarity between our findings and the Jouf University study is that IBS was more prevalent among married students at Jouf University. On the other hand, the number of male students who had a positive history of IBS was higher than the number of female students in the Jouf University study [15].

We found that most of the individuals who participated in this study and reported moderate IBS symptoms also had moderate depression, which demonstrates a significant relationship between IBS and depression. Another study has found that IBS patients were over 20% more likely than non-IBS patients to suffer from depression and anxiety [16]. By contrast, an Indian study has shown that the prevalence of depression in IBS patients was 37.1%, compared to the prevalence in the control group, which was 8.6% [17]. Further studies to confirm the prevalence of both IBS and depression and the nature of their relationship are strongly recommended, especially among high-risk populations.

Additionally, our findings show that 37.11% of the study population confirmed having mild depression, followed by moderate depression in 23.21% of participants. The age of respondents with mild depression mostly ranged between 18 and 30, as did those who were less than 18. Furthermore, mild depression was more prevalent in male and single respondents. Henceforth, we recommend investigating the factors attributed to the prevalence of depression in addition to other psychiatric disorders such as anxiety among the population of colleges and schools. Moreover, suicidal ideation and the risk of suicide should be assessed in those with moderate to severe depression.

Strengths and limitations

In our study, we used a concise and clear survey attached to validated scales to assess the symptoms of depression and IBS. However, the demographic variation could have been minimized to improve the accuracy of the results. Additionally, the number of participants was not completely sufficient to generalize our results because the survey was only conducted in the schools of one region of Saudi Arabia. Another possible limitation, as this was a self-reported, prospective study, is that recall bias is possible. In addition, a significant proportion of the population were diagnosed with other chronic diseases which could contribute to their depression levels. lastly, patients were not questioned about psychiatric medications. henceforth, it is difficult to assess the long-term impact of the treatment of depression on the symptoms of IBS and vice versa.

## Conclusions

Overall, our study indicated that there is an association between different levels of depression and irritable bowel syndrome (IBS) that has been identified among individuals with a positive history of IBS. Most of those who have severe IBS symptoms are found to have moderate and severe depressive symptoms. Moreover, the majority of participants with moderate IBS symptoms exhibited mild and moderate depressive symptoms. Additionally, our findings reported a high prevalence of depression at all levels among the total study population, including respondents with a negative history of IBS. Henceforth, we recommend investigating the factors associated with depression prevalence among the population of schools and colleges. Overall, our study indicated that there is an association between mild depression and IBS that has been identified among individuals with a positive history of IBS. Additionally, our findings reported a high prevalence of depression at all levels among the total study population, including respondents with a negative history of IBS. Henceforth, we recommend investigating the factors associated with depression prevalence among the population of schools and colleges.
